# Establishment and Validation of Pre-Therapy Cervical Vertebrae Muscle Quantification as a Prognostic Marker of Sarcopenia in Patients With Head and Neck Cancer

**DOI:** 10.3389/fonc.2022.812159

**Published:** 2022-02-14

**Authors:** Brennan Olson, Jared Edwards, Catherine Degnin, Nicole Santucci, Michelle Buncke, Jeffrey Hu, Yiyi Chen, Clifton D. Fuller, Mathew Geltzeiler, Aaron J. Grossberg, Daniel Clayburgh

**Affiliations:** ^1^School of Medicine, Oregon Health & Science University, Portland, OR, United States; ^2^Medical Scientist Training Program, Oregon Health & Science University, Portland, OR, United States; ^3^Department of General Surgery, Naval Medical Center San Diego, San Diego, CA, United States; ^4^Biostatistics Shared Resources, Knight Cancer Institute, Oregon Health & Science University, Portland, OR, United States; ^5^Seagen Inc., Bothell, WA, United States; ^6^Department of Radiation Oncology, The University of Texas MD Anderson Cancer Center, Houston, TX, United States; ^7^Department of Otolaryngology/Head and Neck Surgery, Oregon Health & Science University, Portland, OR, United States; ^8^Department of Radiation Medicine, Oregon Health & Science University, Portland, OR, United States; ^9^Brenden-Colson Center for Pancreatic Care, Oregon Health & Science University, Portland, OR, United States; ^10^Cancer Early Detection Advanced Research Center, Knight Cancer Institute, Oregon Health & Science University, Portland, OR, United States; ^11^Operative Care Division, Portland Veterans Affairs Health Care System, Portland, OR, United States

**Keywords:** head and neck cancer, squamous cell carcinoma, surgery, sarcopenia, cachexia, muscle wasting, body composition

## Abstract

**Background:**

Sarcopenia is prognostic for survival in patients with head and neck cancer (HNC). However, identification of this high-risk feature remains challenging without computed tomography (CT) imaging of the abdomen or thorax. Herein, we establish sarcopenia thresholds at the C3 level and determine if C3 sarcopenia is associated with survival in patients with HNC.

**Methods:**

This retrospective cohort study was conducted in consecutive patients with a squamous cell carcinoma of the head and neck with cross-sectional abdominal or neck imaging within 60 days prior to treatment. Measurement of cross-sectional muscle area at L3 and C3 levels was performed from CT imaging. Primary study outcome was overall survival.

**Results:**

Skeletal muscle area at C3 was strongly correlated with the L3 level in both men (n = 188; r = 0.77; p < 0.001) and women (n = 65; r = 0.80; p < 0.001), and C3 sarcopenia thresholds of 14.0 cm^2^/m^2^ (men) and 11.1 cm^2^/m^2^ (women) were best predictive of L3 sarcopenia thresholds. Applying these C3 thresholds to a cohort of patients with neck imaging alone revealed that C3 sarcopenia was independently associated with reduced overall survival in men (HR = 2.63; 95% CI, 1.79, 3.85) but not women (HR = 1.18, 95% CI, 0.76, 1.85).

**Conclusions:**

This study identifies sarcopenia thresholds at the C3 level that best predict L3 sarcopenia in men and women. In HNC, C3-defined sarcopenia is associated with poor survival outcomes in men, but not women, suggesting sarcopenia may differentially affect men and women with HNC.

## Introduction

Patients with cancer frequently experience weight loss, including progressive lean and fat mass catabolism consistent with the paraneoplastic wasting syndrome of cachexia (1, 2). Excessive skeletal muscle wasting, or sarcopenia, is significantly associated with morbidity and mortality for patients with solid tumors (3–5). Sarcopenia can exist as an isolated finding as is often the case in the elderly or secondary to a disease process that activates catabolic programs—including cancer (1, 6). While sarcopenia is richly described as a negative prognostic marker for patients with primary tumors in the abdomen, only recently has its importance been identified in patients with head and neck cancer (HNC) (7–9). Indeed, sarcopenia is a significant predictor of survival and post-operative complications for patients with HNC and shows promise as a risk-stratification tool for patients with this disease (7–10). Furthermore, sarcopenia is significantly associated with the development of cancer-associated fatigue in patients with head and neck cancer, and improvement of muscle mass during disease progression through resistance training may be beneficial in mitigating fatigue and improve overall quality of life (11–13). Therefore, regular detection of sarcopenia in patients with head and neck cancer may allow for early intervention and risk-stratification, which could improve patients’ quality of life and ultimate survival. Despite the clear utility in identifying sarcopenia in patients with HNC, clinical implementation of sarcopenia measures remains challenging due to the lack of established approaches that integrate with standard clinical workflow (14).

In terms of clinical detection, dual-energy x-ray absorptiometry was initially used in detecting sarcopenia, but the most widely utilized method today includes identification of cross-sectional skeletal muscle area from computed tomography (CT) scans (15). However, several alternative methods exist, including bioelectrical impedance analysis, ultrasound of the tibialis anterior, magnetic resonance imaging, and serum biomarker analysis (14, 16–18). Despite the advent of newer detection methods, CT-defined muscle wasting remains highly utilized for several reasons, including its relative ease of analysis compared to alternative methods, and its potential for automation and integration into the electronic health record (19, 20). Since imaging studies (CT or PET/CT) are routinely performed during the clinical workup of HNC, establishing cross-sectional skeletal muscle area thresholds utilizing these scans represent a low-cost, reliable, and reproducible method for determining sarcopenia in this patient population. Current skeletal muscle index (SMI, defined as cross-sectional skeletal muscle area normalized to patients’ body height squared) values for defining sarcopenia are specific to abdominal or lower thoracic musculature (5, 21). As abdominal and lower thoracic imaging is not always performed as part of the HNC workup, only a subset of patients that have imaging studies capturing these regions are included in most previous reports, ultimately introducing selection bias. Therefore, defining and validating sarcopenia thresholds from routinely acquired head and neck images would greatly enhance clinical implementation while allowing better cross-study comparisons. Previous reports demonstrated a significant correlation between cervical and abdominal vertebrae cross sectional muscle area in patients with HNC, suggesting that routine head and neck imaging may be used to identify sarcopenia (22–26). In the present study, we sought to expand upon these findings in a cohort of HNC patients treated with primary surgical resection by defining sex-specific sarcopenia thresholds, evaluating their prognostic value, and validating these associations in an independent patient cohort.

## Methods

### Population Cohort and End Points

We performed a retrospective review by screening medical records of patients who underwent primary surgical resection of head and neck squamous cell carcinoma between January 1, 2005 and December 21, 2017 at Oregon Health and Science University in Portland, Oregon. Electronic health records were reviewed for data collection and included: patient demographics, body mass and height, comorbidities, tumor staging and subsite information, HPV/p16 status, smoking status, treatment information, evidence of recurrence, date and cause of death, and date of last follow up. Patients were classified as underweight (BMI <18.5), normal weight (BMI 18.5-24.9), overweight (BMI 25-29.9) or obese (BMI >30). Smoking status was binned in 3 groups, including never smokers, <10 pack years, ≥10 pack years. Feeding tube classifications included patients never receiving a G-tube, those receiving a G-tube temporary, and those who had feeding tubes *in situ* at the time of last follow up. Tumor sites included: oropharynx, oral cavity, larynx, and other. Pathologic T staging was binned as either T0-2 or T3-4, while pathologic N staging was binned as either N0-1, N2-3, or NX. Charlson Comorbidity Index (CCI) score was calculated as previously described (27) and patients were subsequently stratified as either low risk (CCI <5) or high risk (CCI ≥5). After patient data were abstracted and coupled to their imaging information, all patient data were de-identified for subsequent analyses. This study was approved by the Institutional Review Board at Oregon Health and Science University. Requirement for informed consent was waived due to the retrospective nature of this study.

We utilized two separate cohorts of patients with HNC for analyses. The first cohort, defined as the training cohort, is utilized to establish C3 sarcopenia thresholds and contains patients that have CT imaging that captures both the L3 and C3 vertebral levels. The second cohort, defined as the validation cohort, consists of HNC patients with only neck imaging (skull base to lung apices) and is utilized for validation of these established C3 thresholds on mortality outcomes. Study inclusion for both training and validation cohorts was restricted to patients treated with primary surgical resection of head and neck squamous cell carcinoma at Oregon Health and Science University between January 2005 and December 2017 with PET/CT or CT scans within 60 days prior to surgical resection. Therefore, all patients’ scans utilized in this study were capturing muscle area prior to surgery and/or adjuvant therapy. For the training cohort, patients were required to have a scan that captured both the C3 and L3 vertebral levels, while the validation cohort only required capture of the C3 level.

### Computed Tomography Body Composition Analysis

**Figure 1:**
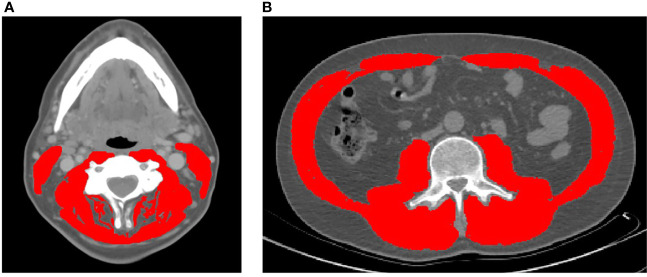
Representative axial CT images of the third cervical **(A)** and third lumbar **(B)** vertebral levels used to quantify skeletal muscle index (highlighted in red).

Body composition analysis of skeletal muscle was performed as previously described in patients with HNC (9, 22). Briefly, the cross-sectional area of skeletal muscle at the center of the third lumbar (L3) and third cervical (C3) vertebrae was determined by segmentation of axial CT images (**Figure 1**). Segmentation analysis was performed using Slice-o-Matic Software (version 5.0; Tomovision; https://www.tomovision.com) to define muscle tissue cross-sectional area. Muscles delineated in the segmentation analysis included the rectus abdominus, abdominal wall, psoas, and the paraspinal muscle groups at the L3 level. Muscles measured at C3 included the paraspinal muscle group and the sternocleidomastoid (SCM). Muscle tissue was defined as -29 to 150 Hounsfield units as described previously (28). The resulting cross-sectional muscle area was then normalized to the square of the patient’s height in meters and used to calculate skeletal muscle index (SMI). At the L3 level, sarcopenia is defined as an SMI of less than 52.4 cm^2^/m^2^ for men and 38.5 cm^2^/m^2^ for women (5). These thresholds are consistent with previous reports in head and neck cancer patients.

### Statistical Analysis

To compare patient and clinical-disease characteristics at baseline, the Chi-square or Fisher’s Exact tests were used for categorical variables; the Wilcoxon rank sum test was used for continuous variables. Data was presented as median (IQR) or frequency (%). Training cohort (n = 253) comparisons were stratified by L3-defined sarcopenia; validation cohort (n = 536) comparisons were stratified by C3-defined sarcopenia.

Correlation was assessed using Pearson’s rho, which assumes normal distribution and a linear relationship between the measurements. Rho greater than 0.8 is considered ‘very strong’ correlation; rho between 0.6-0.79 suggests ‘strong’ correlation. To test for linearity and homoscedasticity we plotted residuals versus fitted values, showing the loess (smoothing) curve in red. Receiver Operating Characteristic (ROC) curves were generated to show the general predictive ability of C3 to predict L3-defined sarcopenia; DeLong’s test of correlated ROC curves was used to discriminate between C3 measurement types (29). We used Youden’s Index to determine the optimal C3 cut-off value for predicting sarcopenia.

Overall survival is the time from initial diagnosis until death by any cause, with participants censored at their last assessment date. Assumptions of proportionality in the survival models were verified graphically and using residual-based models. Univariate and multivariable Cox proportional hazard ratio models were used to assess the risk of death based on demographic and baseline clinical-disease characteristics. We used purposeful selection combined with Bayesian information criterion (BIC) to build the multivariable models, entering all variables from the univariate models with p-value < 0.2. Kaplan-Meier curves with log-rank test were used to display overall survival stratified by C3-defined sarcopenia. All analyses were conducted using R, version 3.5.3.

## Results

### Determination of C3-Defined Sarcopenia Thresholds

The training cohort included patients with both abdominal and neck imaging in order to perform intrapatient correlative analyses of L3 and C3 SMI values to identify appropriate C3 sarcopenia thresholds. Median age of this cohort is 61 (IQR 54, 68) years with 188/253 (74%) patients identifying as male. In this cohort, 6.0% of patients were underweight (BMI <18.5), 32% were normal weight (BMI 18.5-24.9), 38% were overweight (BMI 25-29.9), and 24% were obese (BMI >30). Eighty-nine patients were never smokers (35%), 51 (20%) patients smoked <10 pack years, and 113 (45%) patients had a ≥10 pack year history. Postoperatively, 130 (51%) patients received temporary feeding tubes while 47 (19%) patients still had feeding tubes *in situ* at the time of last follow up. The majority of this cohort had oropharynx disease (147 patients [58%]), while primary tumors in the oral cavity (55 [22%]) and larynx (19 [8%]) were less frequently observed (27). The majority of patients were classified as low-risk by the Charlson Comorbidity Index (CCI <5; 213 [84%]). One hundred three (41%) patients were treated by primary surgical resection alone, while 150 (59%) patients received adjuvant therapy (radiation and/or chemotherapy). Full characteristics and details of this cohort stratified by L3-defined sarcopenia are shown in **eTable 1 in the Supplement**.

**Figure 2:**
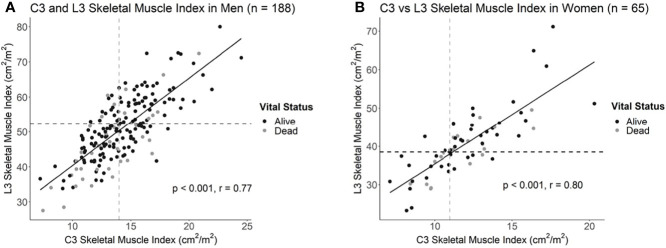
Correlation plots of L3 and C3 skeletal muscle indices in **(A)** men and **(B)** women with head and neck cancer. Dotted lines delineate the intersection of previously established L3 sarcopenia thresholds with the estimated C3 sarcopenia thresholds.

Intrapatient L3 and C3 levels were strongly correlated in both men (n = 188, r = 0.77; p < 0.001) and women (n = 65, r = 0.80; p < 0.001; **Figure 2**; **Supplementary Figure 1**). As it is possible for the borders of the SCM to be obscured by lymph node metastases, we performed comparative analyses amongst L3 and C3 SMI values, both inclusive and exclusive of the SCM, to examine whether inclusion of the SCM improves or worsens predictive capacity of L3-based sarcopenia. Including the SCM in C3 SMI measurements improved predictive capacity of L3-defined sarcopenia in women (AUC = 89.4% vs. 86.3%; p = 0.03), but not men (AUC = 85.9% vs. 85.0%; p = 0.30; **Supplementary Figure 2**). Therefore, we included the SCM in all subsequent analyses. The C3 SMI thresholds with the best model performance based on Youden’s Index were 14.0 cm^2^/m^2^ for men and 11.1 cm^2^/m^2^ for women (**Figure 2**). Finally, we confirmed the utility of C3-defined sarcopenia in this training cohort through survival analysis which demonstrated an association between C3-defined sarcopenia in men (HR = 1.9; 95% CI, 1.2-5.5), but not women (HR = 0.84; 95% CI, 0.30-2.15), with HNC (**Supplementary Figure 3**).

### Survival Analyses in C3-Defined Sarcopenic Patients

We next applied these C3 SMI thresholds to an independent cohort of HNC patients with imaging studies (PET/CT or CT) that captured the head and neck, but not abdomen or lower thorax (**Table 1**). This validation cohort included patients with imaging of the head and neck, but not abdomen or lower thorax. Median age of patients in this cohort was 64 (IQR 56, 72) with 333 patients identifying as male (62%) and 203 identifying as female (38%; **Table 1**). Forty-one (7.7%) patients were underweight, 211 (40%) were normal weight, 164 (31%) were overweight, and 118 (22%) were classified as obese (BMI >30). This cohort included 162 (30%) never smokers, 68 (13%) patients with <10 pack years, and 306 (57%) patients with ≥10 pack years. Postoperatively, 174 (32%) patients received temporary feeding tubes and 105 (20%) patients still had feeding tubes at last follow up. In contrast to the training cohort, the preponderance of this cohort had oral cavity disease (306 patients [57%]), while primary tumors in the oropharynx (118 [22%]) and larynx (79 [15%]) were less frequently observed. Using CCI, 409 [76%] patients were classified as low risk and 127 were classified as [24%] high risk. In the validation cohort, 324 (60%) patients were treated by primary surgical resection alone, with 212 (40%) receiving adjuvant therapy (radiation and/or chemotherapy). Baseline patient characteristics stratified by C3-defined sarcopenia are shown in **Table 1**.

**Table 1:** Clinical-demographic tables for validation set, stratified by C3-defined sarcopenia (Men: C3<14.0; Women: C3<11.1; n = 536).

Risk Factor	Overall, N = 536^1^	Sarcopenic, N = 252^1^	Not sarcopenic, N = 284^1^	p-value^2^
**Age at time of surgery**	64 (56, 72)	65 (58, 71)	62 (55, 72)	0.200
**RT Fractions**	33 (30, 35)	33 (30, 35)	32 (30, 35)	**0.028**
*(Missing)*	344	166	178	
**RT dose**	6,300 (6,000, 6,600)	6,300 (6,000, 6,650)	6,000 (6,000, 6,600)	0.140
*(Missing)*	338	163	175	
**Days from Diagnosis to surgery**	31 (21, 42)	34 (21, 44)	29 (20, 41)	0.090
*(Missing)*	10	6	4	
**Vital Status**				**<0.001**
*Alive*	332 (62%)	134 (53%)	198 (70%)	
*Dead*	204 (38%)	118 (47%)	86 (30%)	
**Sex**				**0.001**
*Male*	333 (62%)	175 (69%)	158 (56%)	
*Female*	203 (38%)	77 (31%)	126 (44%)	
**BMI**				**<0.001**
*Underweight*	41 (8%)	33 (13%)	8 (3%)	
*Normal weight*	211 (40%)	126 (50%)	85 (30%)	
*Overweight*	164 (31%)	71 (28%)	93 (33%)	
*Obese*	118 (22%)	21 (8%)	97 (34%)	
*(Missing)*	2	1	1	
**Smoking Status**				**0.003**
*Never smoke*	162 (30%)	59 (23%)	103 (36%)	
*<10 pack years*	68 (13%)	31 (12%)	37 (13%)	
*>= 10 pack years*	306 (57%)	162 (64%)	144 (51%)	
**Feeding Tube**				0.053
*No G-tube*	257 (48%)	107 (42%)	150 (53%)	
*Temporary G-tube*	174 (32%)	92 (37%)	82 (29%)	
*Permanent G-tube*	105 (20%)	53 (21%)	52 (18%)	
**HPV**				0.055
*HPV-*	99 (57%)	48 (66%)	51 (50%)	
*HPV+*	76 (43%)	25 (34%)	51 (50%)	
*(Missing)*	361	179	182	
**Charlson Comorbidity Score**				0.900
*< 5*	409 (76%)	191 (76%)	218 (77%)	
*>= 5*	127 (24%)	61 (24%)	66 (23%)	
**Tumor site**				0.130
*Oral cavity*	306 (57%)	146 (58%)	160 (56%)	
*Oropharynx*	118 (22%)	48 (19%)	70 (25%)	
*Larynx*	79 (15%)	37 (15%)	42 (15%)	
*Other*	33 (6%)	21 (8%)	12 (4%)	
**Pathologic T category (pT)**				**0.002**
*T0-2*	354 (68%)	149 (61%)	205 (74%)	
*T3-4*	170 (32%)	97 (39%)	73 (26%)	
*(Missing)*	12	6	6	
**Pathologic N category (pN)**				0.500
*N0-1*	348 (66%)	161 (65%)	187 (67%)	
*N2-3*	151 (29%)	71 (29%)	80 (29%)	
*x*	26 (5%)	15 (6%)	11 (4%)	
*(Missing)*	11	5	6	
**Group stage**				**0.024**
*Stage 1*	145 (28%)	60 (24%)	85 (31%)	
*Stage 2*	109 (21%)	42 (17%)	67 (24%)	
*Stage 3*	83 (16%)	43 (17%)	40 (14%)	
*Stage 4*	187 (36%)	101 (41%)	86 (31%)	
*(Missing)*	12	6	6	
**Margins status**				0.500
*Margins-*	424 (84%)	201 (86%)	223 (83%)	
*Margins+*	78 (16%)	33 (14%)	45 (17%)	
*(Missing)*	34	18	16	
**ALI**				0.800
*Absent*	324 (66%)	152 (66%)	172 (67%)	
*Present*	139 (28%)	65 (28%)	74 (29%)	
*Indeterminate/Suspicious*	26 (6%)	14 (6%)	12 (5%)	
*(Missing)*	47	21	26	
**PNI**				0.400
*Absent*	344 (70%)	158 (68%)	186 (72%)	
*Present*	142 (29%)	72 (31%)	70 (27%)	
*Suspicious*	4 (1%)	1 (0.4%)	3 (1%)	
*(Missing)*	46	21	25	
**ENE**				0.300
*Absent*	402 (83%)	185 (81%)	217 (85%)	
*Present*	80 (17%)	43 (19%)	37 (15%)	
*(Missing)*	54	24	30	
**Recurrence**				0.130
*No recurrence*	402 (75%)	181 (72%)	221 (78%)	
*Recurrence*	134 (25%)	71 (28%)	63 (22%)	
**RT**				>0.9
*No RT*	325 (61%)	153 (61%)	172 (61%)	
*RT*	211 (39%)	99 (39%)	112 (39%)	
**Chemotherapy**				0.800
*No Chemo*	437 (82%)	207 (82%)	230 (81%)	
*Chemo*	99 (18%)	45 (18%)	54 (19%)	
**Treatment Group**				>0.9
*Surgery*	324 (60%)	152 (60%)	172 (61%)	
*Surgery + Adjuvant*	212 (40%)	100 (40%)	112 (39%)	

^1^Statistics presented: Median (IQR); n (%).

^2^Statistical tests performed: Wilcoxon rank-sum test; chi-square test of independence; Fisher’s exact test.

Bold text indicates p < .05.

**Figure 3:**
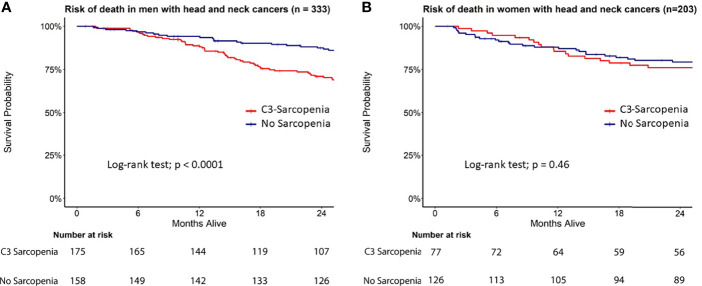
Survival analysis in C3-defined sarcopenic male and female HNC patients. Kaplan Meier curves for **(A)** men and **(B)** women stratified by C3 sarcopenia.

In this cohort, 53% (175/333) of men were classified as sarcopenic compared to just 38% (77/203) of women (**Table 1** and **eTable 2**). Univariate Cox proportional hazards analysis revealed advanced age at time of surgery, C3-defined sarcopenia, underweight BMI status, >10 pack-year smoking status, permanent feeding tube placement, negative HPV status, elevated CCI, pT3-4, pN2-3, group stage 4, angiolymphatic invasion (ALI), perineural invasion (PNI), and extranodal extension (ENE) as significant predictors of survival in men. Univariate cox modelling for variables associated with survival in women included permanent feeding tube placement, elevated CCI, T category (pT3-4), group stage 3 and 4, ALI, and PNI (**eTable3**). Kaplan-Meier survival curves showed a significant difference in survival based on C3-defined sarcopenia for men but not women (**Figure 3**). On multivariable analyses, C3-defined sarcopenia (HR = 2.67; 95% CI, 1.72-4.15), ALI (HR = 2.00; 95% CI, 1.32-3.02), permanent feeding tube placement (HR = 2.33; 95% CI, 1.41-3.88), and age at time of surgery (HR = 1.04; 95% CI, 1.02-1.05) all remained significantly associated with overall survival for men (**Table 2**). In women, PNI (HR = 2.45; 95% CI, 1.48-4.06), ALI (HR = 2.13; 95% CI, 1.22-3.72), and pathologic T category (HR = 1.95; 95% CI, 1.20-3.16) were associated with reduced overall survival on multivariable analysis (**Table 2**). In the training cohort, advanced age was associated with sarcopenia in women, while advanced age was associated with sarcopenia in men in the validation cohort (**eTable4**).

**Table 2:** Multivariable model for factors associated with risk of death with head and neck cancers in validation cohort, by sex (n = 536).

Variables:	Model selected for Men (n = 333)			Model selected for Women (n = 203)		
	HR^1^	95% CI^1^	p-value	HR^1^	95% CI^1^	p-value
**C3-Sarcopenia**			**<0.001**			
*Not sarcopenic*	—	—				
*Sarcopenic*	2.67	1.72, 4.15				
**ALI**			**0.001**			**0.011**
*Absent*	—	—		—	—	
*Present*	2.00	1.32, 3.02		2.13	1.22, 3.72	
**Feeding Tube**			**0.002**			
*No G-tube*	—	—				
*Temporary G-tube*	1.09	0.66, 1.81				
*Permanent G-tube*	2.33	1.41, 3.88				
**Age at time of surgery**	1.04	1.02, 1.05	**<0.001**			
**PNI**						**<0.001**
*Absent*				—	—	
*Present*				2.45	1.48, 4.06	
**Pathologic T category (pT)**						**0.009**
*T0-2*				—	—	
*T3-4*				1.95	1.20, 3.16	

^1^HR, Hazard Ratio; CI, Confidence Interval.

Bold text indicates p < .05.

## Discussion

Skeletal muscle depletion is a well-established prognostic marker in multiple clinical and disease settings, including cancer (7, 8), trauma (30), and drug dose scaling to minimize toxicities (31). Previous reports demonstrate the prognostic utility of sarcopenia is independent of patient body mass (5, 8, 32), and that height and weight formulae are not sufficient to capture sarcopenia (33). However, identification of this high-risk disease feature is challenging in HNC because imaging studies that capture the abdomen or lower thorax are infrequent in this population. Although previous studies demonstrate promise in utilizing neck imaging as a marker of sarcopenia (22, 25, 26, 34), we sought to expand upon these foundational studies by putting forth normalized sex-specific sarcopenia thresholds and testing their prognostic utility in an independent cohort of patients with neck imaging alone. To our knowledge, our study is the first to establish normalized sex-specific C3 sarcopenia thresholds and validate their prognostic value in an independent cohort of HNC patients with imaging studies specific to the neck. We found that these C3 SMI thresholds were prognostic in men, but not women, with HNC.

Several foundational studies demonstrated a strong correlation of C3 and L3 skeletal muscle area in patients with HNC consistent with our findings herein (22, 23, 25, 35–37). One report utilized a single SMI cut-off for both men and women, an approach that may not accurately account for intrinsic differences in body habitus between male and female sexes (36). A recent study by Ufuk and colleagues found that among the C2-C4 vertebral levels, the paraspinal muscles at C3 were the most predictive of L3 in males, while summative C2-C4 SCM measurements were the most discriminative in females (23). However, a combinatorial approach in which both paraspinal and SCM muscle groups was not utilized in this report, and it stands to reason that increasing the measurable skeletal muscle area at the cervical level would only serve to increase the predictive capacity for sarcopenia at the lumbar level, as our data herein suggest (**Supplementary Figure 2**). Another recent report investigating the predictive capacity of C3 SMI on L3 SMI in a Korean HNC population demonstrated significant correlation in non-sarcopenic patients, but no such correlation in sarcopenic patients (defined as a calculated L3 muscle index of <55 cm^2^/m^2^ in men and <39 cm^2^/m^2^ in women) (38). In the present study, we observe significant correlation for both non-sarcopenic and sarcopenic HNC patients. Possible explanations for these differences in results could be due to the population of study or techniques in measuring skeletal muscle area (38). Specifically, we included both paraspinal and SCM muscles when measuring C3 SMI due to its improved predictive capacity of L3 (**Supplementary Figure 2**); we also utilized previously published L3 sarcopenia thresholds of <52.4 cm^2^/m^2^ for men and <38.5 cm^2^/m^2^ for women (23, 38). Similarly, in another recent report utilizing cross-sectional muscle area at the C3 level to predict L3 cross-sectional area, the authors classified sarcopenia as the lowest quartile of predicted L3 muscle area, while the work herein sought to base C3 sarcopenia thresholds on L3 sarcopenia thresholds that are validated across multiple cancers and conditions (25). Accordingly, it is possible that these C3 sarcopenia thresholds extend to other diseases and clinical settings in which the abdomen or lower thorax are not routinely captured by CT imaging, such as neurologic cancers. However, identifying the most suitable sarcopenia thresholds appears to be multifactorial and dependent on sex and disease-specific conditions (39), further highlighting the need for validating sarcopenia thresholds in a context-specific manner.

In the context of this recent literature, we believe our work adds substantially to this area of research for several reasons: (1) through the largest cohort study to date, we further evidence that muscle area at the C3 vertebral level is closely predictive of the L3 level in both sarcopenic and non-sarcopenic HNC patients; (2) we establish that measuring both paraspinal and SCM musculature is more predictive than paraspinal SMI calculations alone, as many previous studies utilize only paraspinal muscles for sarcopenia measurements; (3) we establish normalized sex-specific C3 thresholds that are best-predictive of widely published L3 sarcopenia thresholds, rather than utilizing *ad hoc* thresholds that are predictive of a single cohort; (4) we validate the prognostic utility of our proposed C3 sarcopenia thresholds in an independent cohort of patients with only neck CT imaging available; and (5) finally, we are the first to demonstrate a sexually-dimorphic survival outcome for HNC patients with sarcopenia, as sarcopenia was associated with reduced survival in men, but not women (25, 39). To our knowledge, this is the largest dataset of women with HNC stratified by sarcopenia, and the first to individually assess sex-specific survival outcomes during this disease.

Like many research areas, sarcopenia has largely been studied in males in both murine and clinical settings, while few studies evaluating sex as a biological variable exist (40, 41). Amongst experimental rodent models of colon- and HNC-associated cachexia, male mice lost a larger proportion of lean mass compared to female mice with similar disease burden (42, 43). Several studies also report a significantly lower prevalence of sarcopenia in women with various cancers, including non-small cell lung and gastrointestinal cancers (44, 45). In the present study, we observe 55% of men are sarcopenic at the time of diagnosis compared to just 39% of women (p < 0.001). In addition to reports that describe a decrease in the prevalence of sarcopenia in women at the time of cancer diagnosis, Kilgour and colleagues showed a strong association between muscle mass and cancer-associated fatigue in men, but not in women (46). Burkart and colleagues recently showed an association between sarcopenia and overall survival in men with aggressive B-cell Non-Hodgkin Lymphoma, while women demonstrated no such association (47). Conversely, sarcopenia was associated with poorer survival outcomes in women with non-metastatic breast cancer (32). These studies and more suggest that sarcopenia may not only disproportionately appear between men and women during disease, but also differentially influence quality of life and mortality in a sex- and disease-specific manner (39).

It is plausible that the sarcopenia observed in this population is associated with cachexia, a disease-associated metabolic syndrome that significantly reduces patient’s quality of life and ultimate survival (1). In the present study, it is possible that sarcopenia portends the development of cachexia more frequently in men than women, or that muscle loss is not associated with impaired resilience in women with HNC—future prospective investigations are needed to test these hypotheses. While this study demonstrates pre-therapy C3-defined sarcopenia is a useful prognostic marker in men, but not women, diagnosed with HNC, a recent report demonstrated that conversion to sarcopenia (detected by L3 muscle area) was associated with a reduction in overall survival in patients with HNC treated with definitive RT (7). Therefore, the sex-specific influence of post-therapy conversion to sarcopenia using the C3-sarcopenia thresholds established herein remains an area of active investigation. Collectively, our findings highlight the need for future studies to be deliberate in examining sex-specific effects and prevalence of sarcopenia.

### Limitations

This study has several limitations that should be taken into consideration when interpreting these data. As with any retrospective review, this study is subjected to missing data and heterogeneous patient follow-up. This study was performed at a single tertiary care institution, resulting in a patient demographic that may not be reflective of other areas of the country. Finally, given the timeframe of this study that traverses the recognition of HPV/p16 as a prognostic factor, these patients are staged by the American Joint Committee on Cancer (AJCC) seventh edition, as opposed AJCC eight edition staging criteria.

C3 thresholds were not developed against the ‘gold standard’ for sarcopenia, either volumetric full body cross sectional imaging or dual x-ray absorptiometry. A limitation of this study is that a surrogate marker of sarcopenia, normalized L3 skeletal muscle cross sectional area, is used to estimate the C3 threshold value and may imprecisely estimate sarcopenia. However, this measure is associated with survival endpoints in multiple disease states, supporting its use in the present study. We directly tested C3 measurements with risk of death using a partitioning approach (partDSA survival package in R) which generated a similar threshold value for men but provided no distinct C3 cut-off value for women.

## Conclusions

Taken together, this study demonstrates that C3-sarcopenia thresholds are strongly associated with previously defined L3 sarcopenia thresholds, and C3-defined sarcopenia is independently associated with reduced survival in men, but not women with HNC. We propose that the C3-defined sarcopenia thresholds herein represent a useful prognostic tool for men with HNC. Future research concerning these observations is warranted, including determining whether these thresholds and sex-specific survival associations extend to other pathologies.

## Data Availability Statement

The raw data supporting the conclusions of this article will be made available by the authors, without undue reservation.

## Ethics Statement

This study was approved by the Institutional Review Board at Oregon Health and Science University. Written informed consent for participation was not required for this study in accordance with the national legislation and the institutional requirements.

## Author Contributions

Conceptualization: BO, AG, and DC. Data curation: BO, JE, NS, MB, and JH. Formal analysis: CD and YC. Funding acquisition: BO and AG. Investigation: BO, CDF, MG, AG, and DC. Methodology: BO, JE, CD, and YC. Resources: BO, CD, CDF, MG, AG, and DC. Software: BO and AG. Supervision: CDF, MG, AG, and DC. Writing - original draft: BO, AG, and DC. Writing - review and editing: BO, JE, CD, NS, MB, JH, YC, CDF, MG, AG, and DC. All authors contributed to the article and approved the submitted version.

## Conflict of Interest

Author YC is currently employed by Seagen Inc, although this employment resulted after data and analyses were completed.

The remaining authors declare that the research was conducted in the absence of any commercial or financial relationships that could be construed as a potential conflict of interest.

## Publisher’s Note

All claims expressed in this article are solely those of the authors and do not necessarily represent those of their affiliated organizations, or those of the publisher, the editors and the reviewers. Any product that may be evaluated in this article, or claim that may be made by its manufacturer, is not guaranteed or endorsed by the publisher.

## References

[1] Olson B Marks DL Grossberg AJ. Diverging Metabolic Programmes and Behaviours During States of Starvation, Protein Malnutrition, and Cachexia. J Cachexia Sarcopenia Muscle (2020) 11(6):1429–46. doi: 10.1002/jcsm.12630 PMC7749623 32985801

[2] Baracos VE Martin L Korc M Guttridge DC Fearon KCH. Cancer-Associated Cachexia. Nat Rev Dis Primers (2018) 4:17105. doi: 10.1038/nrdp.2017.105 29345251

[3] Rinninella E Cintoni M Raoul P Pozzo C Strippoli A Bria E. Muscle Mass, Assessed at Diagnosis by L3-CT Scan as a Prognostic Marker of Clinical Outcomes in Patients With Gastric Cancer: A Systematic Review and Meta-Analysis. Clin Nutr (2019) 39(7):2045–54. doi: 10.1016/j.clnu.2019.10.021 31718876

[4] Shachar SS Williams GR Muss HB Nishijima TF. Prognostic Value of Sarcopenia in Adults With Solid Tumours: A Meta-Analysis and Systematic Review. Eur J Cancer (Oxf Engl 1990) (2016) 57:58–67. doi: 10.1016/j.ejca.2015.12.030 26882087

[5] Prado CM Lieffers JR McCargar LJ Reiman T Sawyer MB Martin L. Prevalence and Clinical Implications of Sarcopenic Obesity in Patients With Solid Tumours of the Respiratory and Gastrointestinal Tracts: A Population-Based Study. Lancet Oncol (2008) 9(7):629–35. doi: 10.1016/S1470-2045(08)70153-0 18539529

[6] Cruz-Jentoft AJ Bahat G Bauer J Boirie Y Bruyère O Cederholm T. Sarcopenia: Revised European Consensus on Definition and Diagnosis. Age Ageing (2019) 48(1):16–31. doi: 10.1093/ageing/afy169 30312372 PMC6322506

[7] Grossberg AJ Chamchod S Fuller CD Mohamed AS Heukelom J Eichelberger H. Association of Body Composition With Survival and Locoregional Control of Radiotherapy-Treated Head and Neck Squamous Cell Carcinoma. JAMA Oncol (2016) 2(6):782–9. doi: 10.1001/jamaoncol.2015.6339 PMC5080910 26891703

[8] Stone L Olson B Mowery A Krasnow S Jiang A Li R. Association Between Sarcopenia and Mortality in Patients Undergoing Surgical Excision of Head and Neck Cancer. JAMA Otolaryngol Head Neck Surg (2019) 145(7):647–54. doi: 10.1001/jamaoto.2019.1185 PMC6555480 31169874

[9] Olson B Edwards J Stone L Jiang A Zhu X Holland J. Association of Sarcopenia With Oncologic Outcomes of Primary Surgery or Definitive Radiotherapy Among Patients With Localized Oropharyngeal Squamous Cell Carcinoma. JAMA Otolaryngol Head Neck Surg (2020) 146(8):714–22. doi: 10.1001/jamaoto.2020.1154 PMC7290710 32525518

[10] Achim V Bash J Mowery A Guimaraes AR Li R Schindler J. Prognostic Indication of Sarcopenia for Wound Complication After Total Laryngectomy. JAMA Otolaryngol Head Neck Surg (2017) 143(12):1159–65. doi: 10.1001/jamaoto.2017.0547 28448668

[11] Grote M Maihöfer C Weigl M Davies-Knorr P Belka C. Progressive Resistance Training in Cachectic Head and Neck Cancer Patients Undergoing Radiotherapy: A Randomized Controlled Pilot Feasibility Trial. Radiat Oncol (2018) 13(1):215. doi: 10.1186/s13014-018-1157-0 30400971 PMC6219249

[12] Sandmael JA Bye A Solheim TS Stene GB Thorsen L Kaasa S. Feasibility and Preliminary Effects of Resistance Training and Nutritional Supplements During Versus After Radiotherapy in Patients With Head and Neck Cancer: A Pilot Randomized Trial. Cancer (2017) 123(22):4440–8. doi: 10.1002/cncr.30901 28759113

[13] Lønbro S Dalgas U Primdahl H Johansen J Nielsen JL Aagaard P. Progressive Resistance Training Rebuilds Lean Body Mass in Head and Neck Cancer Patients After Radiotherapy–Results From the Randomized DAHANCA 25B Trial. Radiother Oncol (2013) 108(2):314–9. doi: 10.1016/j.radonc.2013.07.002 23932192

[14] Grossberg AJ Rock CD Edwards J Mohamed ASR Ruzensky D Currie A. Bioelectrical Impedance Analysis as a Quantitative Measure of Sarcopenia in Head and Neck Cancer Patients Treated With Radiotherapy. Radiother Oncol (2021) 159:21–7. doi: 10.1016/j.radonc.2021.03.005 PMC8205950 33736997

[15] Petak S Barbu CG Yu EW Fielding R Mulligan K Sabowitz B. The Official Positions of the International Society for Clinical Densitometry: Body Composition Analysis Reporting. J Clin Densitom (2013) 16(4):508–19. doi: 10.1016/j.jocd.2013.08.018 24183640

[16] Leigheb M de Sire A Colangelo M Zagaria D Grassi FA Rena O. Sarcopenia Diagnosis: Reliability of the Ultrasound Assessment of the Tibialis Anterior Muscle as an Alternative Evaluation Tool. Diagn (Basel Switzerland) (2021) 11(11):2158. doi: 10.3390/diagnostics11112158 PMC8624824 34829505

[17] de Sire A Baricich A Renò F Cisari C Fusco N Invernizzi M. Myostatin as a Potential Biomarker to Monitor Sarcopenia in Hip Fracture Patients Undergoing a Multidisciplinary Rehabilitation and Nutritional Treatment: A Preliminary Study. Aging Clin Exp Res (2020) 32(5):959–62. doi: 10.1007/s40520-019-01436-8 31838642

[18] Erlandson MC Lorbergs AL Mathur S Cheung AM. Muscle Analysis Using pQCT, DXA and MRI. Eur J Radiol (2016) 85(8):1505–11. doi: 10.1016/j.ejrad.2016.03.001 27005009

[19] Amarasinghe KC Lopes J Beraldo J Kiss N Bucknell N Everitt S. A Deep Learning Model to Automate Skeletal Muscle Area Measurement on Computed Tomography Images. Front Oncol (2021) 11:580806. doi: 10.3389/fonc.2021.580806 34026597 PMC8138051

[20] Paris MT Tandon P Heyland DK Furberg H Premji T Low G. Automated Body Composition Analysis of Clinically Acquired Computed Tomography Scans Using Neural Networks. Clin Nutr (2020) 39(10):3049–55. doi: 10.1016/j.clnu.2020.01.008 PMC7374050 32007318

[21] Kaplan SJ Zhao KL Koren M Bentov I Reed MJ Pham TN. Thresholds and Mortality Associations of Paraspinous Muscle Sarcopenia in Older Trauma Patients. JAMA Surg (2020) 155(7):662–4. doi: 10.1001/jamasurg.2020.0435 PMC7191436 32347915

[22] Swartz JE Pothen AJ Wegner I Smid EJ Swart KM de Bree R. Feasibility of Using Head and Neck CT Imaging to Assess Skeletal Muscle Mass in Head and Neck Cancer Patients. Oral Oncol (2016) 62:28–33. doi: 10.1016/j.oraloncology.2016.09.006 27865369

[23] Ufuk F Herek D Yüksel D. Diagnosis of Sarcopenia in Head and Neck Computed Tomography: Cervical Muscle Mass as a Strong Indicator of Sarcopenia. Clin Exp Otorhinolaryngol (2019) 12(3):317–24. doi: 10.21053/ceo.2018.01613 PMC6635710 30947498

[24] Bril SI Wendrich AW Swartz JE Wegner I Pameijer F Smid EJ. Interobserver Agreement of Skeletal Muscle Mass Measurement on Head and Neck CT Imaging at the Level of the Third Cervical Vertebra. Eur Arch Otorhinolaryngol (2019) 276(4):1175–82. doi: 10.1007/s00405-019-05307-w PMC6426814 30689037

[25] van Rijn-Dekker MI van den Bosch L van den Hoek JGM Bijl HP van Aken ESM van der Hoorn A. Impact of Sarcopenia on Survival and Late Toxicity in Head and Neck Cancer Patients Treated With Radiotherapy. Radiother Oncol (2020) 147:103–10. doi: 10.1016/j.radonc.2020.03.014 32251949

[26] Yoshimura T Suzuki H Takayama H Higashi S Hirano Y Tezuka M. Prognostic Role of Preoperative Sarcopenia Evaluation of Cervical Muscles With Long-Term Outcomes of Patients With Oral Squamous Cell Carcinoma. Cancers (Basel) (2021) 13(18):4725. doi: 10.3390/cancers13184725 34572952 PMC8465585

[27] Charlson ME Pompei P Ales KL MacKenzie CR. A New Method of Classifying Prognostic Comorbidity in Longitudinal Studies: Development and Validation. J Chronic Dis (1987) 40(5):373–83. doi: 10.1016/0021-9681(87)90171-8 3558716

[28] Mitsiopoulos N Baumgartner RN Heymsfield SB Lyons W Gallagher D Ross R. Cadaver Validation of Skeletal Muscle Measurement by Magnetic Resonance Imaging and Computerized Tomography. J Appl Physiol (Bethesda Md 1985) (1998) 85(1):115–22. doi: 10.1152/jappl.1998.85.1.115 9655763

[29] DeLong ER DeLong DM Clarke-Pearson DL. Comparing the Areas Under Two or More Correlated Receiver Operating Characteristic Curves: A Nonparametric Approach. Biometrics (1988) 44(3):837–45. doi: 10.2307/2531595 3203132

[30] Tanabe C Reed MJ Pham TN Penn K Bentov I Kaplan SJ. Association of Brain Atrophy and Masseter Sarcopenia With 1-Year Mortality in Older Trauma Patients. JAMA Surg (2019) 154(8):716–23. doi: 10.1001/jamasurg.2019.0988 PMC6506900 31066880

[31] Bozzetti F. Forcing the Vicious Circle: Sarcopenia Increases Toxicity, Decreases Response to Chemotherapy and Worsens With Chemotherapy. Ann Oncol (2017) 28(9):2107–18. doi: 10.1093/annonc/mdx271 28911059

[32] Caan BJ Cespedes Feliciano EM Prado CM Alexeeff S Kroenke CH Bradshaw P. Association of Muscle and Adiposity Measured by Computed Tomography With Survival in Patients With Nonmetastatic Breast Cancer. JAMA Oncol (2018) 4(6):798–804. doi: 10.1001/jamaoncol.2018.0137 29621380 PMC6584322

[33] Chamchod S Fuller CD Mohamed AS Grossberg A Messer JA Heukelom J. Quantitative Body Mass Characterization Before and After Head and Neck Cancer Radiotherapy: A Challenge of Height-Weight Formulae Using Computed Tomography Measurement. Oral Oncol (2016) 61:62–9. doi: 10.1016/j.oraloncology.2016.08.012 PMC5079277 27688106

[34] Jung AR Roh JL Kim JS Choi SH Nam SY Kim SY. Efficacy of Head and Neck Computed Tomography for Skeletal Muscle Mass Estimation in Patients With Head and Neck Cancer. Oral Oncol (2019) 95:95–9. doi: 10.1016/j.oraloncology.2019.06.009 31345401

[35] Muresan BT Sánchez Juan C Artero A Hernández Machancoses A Almendros-Blanco P Montoro A. Diagnosis of Pre-Sarcopenia From a Single Selectional Crosscut at C3 Region, Using CT Scans Before Radiotherapy. Nutricion Hospitalaria (2019) 36(5):1101–8. doi: 10.20960/nh.02422 31475837

[36] Bril SI Chargi N Wendrich AW Wegner I Bol GH Smid EJ. Validation of Skeletal Muscle Mass Assessment at the Level of the Third Cervical Vertebra in Patients With Head and Neck Cancer. Oral Oncol (2021) 123:105617. doi: 10.1016/j.oraloncology.2021.105617 34749251

[37] Bril SI van Beers MA Chargi N Carrillo Minulina N Smid EJ Dankbaar JW. Skeletal Muscle Mass at C3 is a Strong Predictor for Skeletal Muscle Mass at L3 in Sarcopenic and Non-Sarcopenic Patients With Head and Neck Cancer. Oral Oncol (2021) 122:105558. doi: 10.1016/j.oraloncology.2021.105558 34627078

[38] Yoon JK Jang JY An YS Lee SJ. Skeletal Muscle Mass at C3 may Not be a Strong Predictor for Skeletal Muscle Mass at L3 in Sarcopenic Patients With Head and Neck Cancer. PloS One (2021) 16(7):e0254844. doi: 10.1371/journal.pone.0254844 34280248 PMC8289025

[39] Bauer J Morley JE Schols A Ferrucci L Cruz-Jentoft AJ Dent E. Sarcopenia: A Time for Action. SCWD Position Paper J Cachexia Sarcopenia Muscle (2019) 10(5):956–61. doi: 10.1002/jcsm.12483 PMC6818450 31523937

[40] Montalvo RN Counts BR Carson JA. Understanding Sex Differences in the Regulation of Cancer-Induced Muscle Wasting. Curr Opin Support Palliat Care (2018) 12(4):394–403. doi: 10.1097/SPC.0000000000000380 30102621 PMC6239206

[41] Zhong X Zimmers TA. Sex Differences in Cancer Cachexia. Curr Osteoporos Rep (2020) 18(6):646–54. doi: 10.1007/s11914-020-00628-w PMC7732790 33044689

[42] Hetzler KL Hardee JP Puppa MJ Narsale AA Sato S Davis JM. Sex Differences in the Relationship of IL-6 Signaling to Cancer Cachexia Progression. Biochim Biophys Acta (2015) 1852(5):816–25. doi: 10.1016/j.bbadis.2014.12.015 PMC4372501 25555992

[43] Olson B Norgard MA Levasseur PR Zhu X Marks DL. Physiologic and Molecular Characterization of a Novel Murine Model of Metastatic Head and Neck Cancer Cachexia. J Cachexia Sarcopenia Muscle (2021) 12(5):1312–32. doi: 10.1002/jcsm.12745 PMC8517353 34231343

[44] Baracos VE Reiman T Mourtzakis M Gioulbasanis I Antoun S. Body Composition in Patients With Non-Small Cell Lung Cancer: A Contemporary View of Cancer Cachexia With the Use of Computed Tomography Image Analysis. Am J Clin Nutr (2010) 91(4):1133s–7s. doi: 10.3945/ajcn.2010.28608C 20164322

[45] Anoveros-Barrera A Bhullar AS Stretch C Esfandiari N Dunichand-Hoedl AR Martins KJB. Clinical and Biological Characterization of Skeletal Muscle Tissue Biopsies of Surgical Cancer Patients. J Cachexia Sarcopenia Muscle (2019) 10(6):1356–77. doi: 10.1002/jcsm.12466 PMC9536086 31307124

[46] Kilgour RD Vigano A Trutschnigg B Hornby L Lucar E Bacon SL. Cancer-Related Fatigue: The Impact of Skeletal Muscle Mass and Strength in Patients With Advanced Cancer. J Cachexia Sarcopenia Muscle (2010) 1(2):177–85. doi: 10.1007/s13539-010-0016-0 PMC3060645 21475694

[47] Burkart M Schieber M Basu S Shah P Venugopal P Borgia JA. Evaluation of the Impact of Cachexia on Clinical Outcomes in Aggressive Lymphoma. Br J Haematol (2019) 186(1):45–53. doi: 10.1111/bjh.15889 30941741

