# Supplementary material for: Establishment and Validation of Pre-Therapy Cervical Vertebrae Muscle Quantification as a Prognostic Marker of Sarcopenia in Patients With Head and Neck Cancer
Source: Front Oncol. 2022 Feb 14;12:812159. doi: 10.3389/fonc.2022.812159 (PMC8882831; doi:10.3389/fonc.2022.812159)

Supplementary Online Content

eTable 1. Baseline Patient Characteristics of OHSU patients with imaging capturing the abdomen and neck, training cohort (men, n = 188, women, n = 65).

eTable 2. Body Composition and C3-defined sarcopenia status of OHSU patients with imaging capturing only the neck stratified by sex, validation cohort (men, n = 333; women, n=203)

eTable 3. Univariate Cox Models for variables associated with risk of death with head and neck cancers, by sex, validation cohort (men, n = 333; women, n=203). Reports Wald p-value.

eTable 4. Association between age (continuous) and sarcopenia by sex for both training and validation cohorts.

eTable 1.

| **Variables** | **Overall, N = 253^1^** | **Sarcopenia, N = 140^1^** | **Not sarcopenic, N = 113^1^** | **p-value^2^** |
| --- | --- | --- | --- | --- |
| **Age** | 61 (54, 68) | 63 (58, 71) | 57 (52, 63) | **<0.001** |
| **RT Fractions** | 32 (30, 35) | 32 (30, 35) | 33 (30, 35) | 0.900 |
| *(Missing)* | 147 | 83 | 64 |  |
| **RT dose** | 6,300 (6,000, 6,400) | 6,300 (6,000, 6,400) | 6,060 (6,000, 6,532) | 0.700 |
| *(Missing)* | 123 | 70 | 53 |  |
| **C3-Sarcopenia** |  |  |  | **<0.001** |
| *Sarcopenia* | 124 (49%) | 105 (75%) | 19 (17%) |  |
| *No sarcopenia* | 129 (51%) | 35 (25%) | 94 (83%) |  |
| **Vital Status** |  |  |  | **0.046** |
| *Alive* | 192 (76%) | 99 (71%) | 93 (82%) |  |
| *Dead* | 61 (24%) | 41 (29%) | 20 (18%) |  |
| **Sex** |  |  |  | **0.031** |
| *Male* | 188 (74%) | 112 (80%) | 76 (67%) |  |
| *Female* | 65 (26%) | 28 (20%) | 37 (33%) |  |
| **BMI** |  |  |  | **<0.001** |
| *Underweight* | 15 (6.0%) | 14 (10%) | 1 (0.9%) |  |
| *Normal weight* | 82 (32%) | 62 (45%) | 20 (18%) |  |
| *Overweight* | 95 (38%) | 49 (35%) | 46 (41%) |  |
| *Obese* | 60 (24%) | 14 (10%) | 46 (41%) |  |
| *(Missing)* | 1 | 1 | 0 |  |
| **Smoking Status** |  |  |  | **0.003** |
| *Never smoke* | 89 (35%) | 40 (29%) | 49 (43%) |  |
| *<10 pack years* | 51 (20%) | 24 (17%) | 27 (24%) |  |
| *>= 10 pack years* | 113 (45%) | 76 (54%) | 37 (33%) |  |
| **Feeding Tube** |  |  |  | 0.130 |
| *No G-tube* | 76 (30%) | 38 (27%) | 38 (34%) |  |
| *Temporary G-tube* | 130 (51%) | 70 (50%) | 60 (53%) |  |
| *Permanent G-tube* | 47 (19%) | 32 (23%) | 15 (13%) |  |
| **HPV** |  |  |  | 0.120 |
| *HPV-* | 33 (27%) | 22 (33%) | 11 (19%) |  |
| *HPV+* | 90 (73%) | 44 (67%) | 46 (81%) |  |
| *(Missing)* | 130 | 74 | 56 |  |
| **Tumor site** |  |  |  | 0.130 |
| *Oral cavity* | 55 (22%) | 37 (27%) | 18 (16%) |  |
| *Oropharynx* | 147 (58%) | 76 (55%) | 71 (63%) |  |
| *Larynx* | 19 (8%) | 12 (9%) | 7 (6%) |  |
| *Other* | 31 (12%) | 14 (10%) | 17 (15%) |  |
| *(Missing)* | 1 | 1 | 0 |  |
| **Charlson Comorbidity Score** |  |  |  | **0.027** |
| *< 5* | 213 (84%) | 111 (79%) | 102 (90%) |  |
| *>= 5* | 40 (16%) | 29 (21%) | 11 (9.7%) |  |
| **Pathologic T category (pT)** |  |  |  | 0.300 |
| *T0-2* | 175 (70%) | 92 (67%) | 83 (73%) |  |
| *T3-4* | 76 (30%) | 46 (33%) | 30 (27%) |  |
| *(Missing)* | 2 | 2 | 0 |  |
| **Pathologic N category (pN)** |  |  |  | >0.9 |
| *N0-1* | 140 (56%) | 77 (56%) | 63 (56%) |  |
| *N2-3* | 111 (44%) | 61 (44%) | 50 (44%) |  |
| *(Missing)* | 2 | 2 | 0 |  |
| **Group stage** |  |  |  | 0.400 |
| *Stage 1* | 59 (24%) | 28 (20%) | 31 (27%) |  |
| *Stage 2* | 78 (31%) | 43 (31%) | 35 (31%) |  |
| *Stage 3* | 26 (10%) | 13 (9.4%) | 13 (12%) |  |
| *Stage 4* | 88 (35%) | 54 (39%) | 34 (30%) |  |
| *(Missing)* | 2 | 2 | 0 |  |
| **Margins status** |  |  |  | >0.9 |
| *Negative* | 194 (80%) | 108 (79%) | 86 (80%) |  |
| *Positive* | 50 (20%) | 28 (21%) | 22 (20%) |  |
| *(Missing)* | 9 | 4 | 5 |  |
| **ALI** |  |  |  | 0.400 |
| *Absent* | 119 (49%) | 62 (46%) | 57 (53%) |  |
| *Present* | 123 (51%) | 72 (54%) | 51 (47%) |  |
| *(Missing)* | 11 | 6 | 5 |  |
| **PNI** |  |  |  | 0.400 |
| *Absent* | 161 (66%) | 86 (64%) | 75 (69%) |  |
| *Present* | 82 (34%) | 49 (36%) | 33 (31%) |  |
| *(Missing)* | 10 | 5 | 5 |  |
| **ENE** |  |  |  | >0.9 |
| *Absent* | 167 (69%) | 92 (69%) | 75 (69%) |  |
| *Present* | 75 (31%) | 41 (31%) | 34 (31%) |  |
| *(Missing)* | 11 | 7 | 4 |  |
| **RT** |  |  |  | 0.400 |
| *No RT* | 105 (42%) | 62 (44%) | 43 (38%) |  |
| *RT* | 148 (58%) | 78 (56%) | 70 (62%) |  |
| **Treatment group** |  |  |  | 0.400 |
| *Surgery* | 103 (41%) | 61 (44%) | 42 (37%) |  |
| *Surgery + adjuvant* | 150 (59%) | 79 (56%) | 71 (63%) |  |
| **Recurrence** |  |  |  | 0.300 |
| *No recurrence* | 195 (77%) | 104 (74%) | 91 (81%) |  |
| *Recurrence* | 58 (23%) | 36 (26%) | 22 (19%) |  |
| ^1^Statistics presented: Median (IQR); n (%) | | | | |
| ^2^Statistical tests performed: Wilcoxon rank-sum test; chi-square test of independence | | | | |

eTable 2.

| **Risk Factor** | **Overall, N = 536^1^** | **Male, N = 333^1^** | **Female, N = 203^1^** | **p-value^2^** |
| --- | --- | --- | --- | --- |
| **BMI** |  |  |  | 0.140 |
| *Underweight* | 41 (7.7%) | 20 (6.0%) | 21 (10%) |  |
| *Normal weight* | 211 (40%) | 129 (39%) | 82 (40%) |  |
| *Overweight* | 164 (31%) | 111 (34%) | 53 (26%) |  |
| *Obese* | 118 (22%) | 71 (21%) | 47 (23%) |  |
| *(Missing)* | 2 | 2 | 0 |  |
| **C3-Sarcopenia** |  |  |  | **0.001** |
| *Sarcopenic* | 252 (47%) | 175 (53%) | 77 (38%) |  |
| *Not sarcopenic* | 284 (53%) | 158 (47%) | 126 (62%) |  |
| **C3 SMI+SCM (cm^2^/m^2^)** | 13.2 (11.5, 15.1) | 13.8 (12.3, 15.8) | 12.0 (10.3, 13.8) | **<0.001** |

eTable 3.

|  | **Univariate Models for Men (n = 333)** | | | | | **Univariate Models for Women (n = 203)** | | | | |
| --- | --- | --- | --- | --- | --- | --- | --- | --- | --- | --- |
| **Risk Factor** | **N** | **Event N** | **HR^1^** | **95% CI^1^** | **p-value** | **N** | **Event N** | **HR^1^** | **95% CI^1^** | **p-value** |
| **Age at time of surgery** | 333 | 123 | 1.04 | 1.02, 1.05 | **<0.001** | 203 | 80 | 1.00 | 0.99, 1.02 | 0.651 |
| **RT Fractions** | 143 | 50 | 1.00 | 1.00, 1.01 | 0.284 | 49 | 19 | 1.00 | 0.99, 1.01 | 0.826 |
| **RT dose** | 146 | 51 | 1.00 | 1.00, 1.00 | 0.492 | 52 | 20 | 1.00 | 1.00, 1.00 | 0.358 |
| **Days from Diagnosis to surgery** | 325 | 118 | 1.00 | 0.99, 1.01 | 0.654 | 201 | 79 | 1.00 | 0.99, 1.01 | 0.457 |
| **C3-Sarcopenia** |  | 123 |  |  | **<0.001** |  | 80 |  |  | 0.465 |
| Not sarcopenic | 158 |  | — | — |  | 126 |  | — | — |  |
| Sarcopenic | 175 |  | 2.63 | 1.79, 3.85 |  | 77 |  | 1.18 | 0.76, 1.85 |  |
| **BMI** |  | 123 |  |  | **<0.001** |  | 80 |  |  | 0.16 |
| Normal weight | 129 |  | — | — |  | 82 |  | — | — |  |
| Underweight | 20 |  | 1.87 | 1.00, 3.49 |  | 21 |  | 1.68 | 0.87, 3.25 |  |
| Overweight | 111 |  | 0.57 | 0.37, 0.86 |  | 53 |  | 0.72 | 0.40, 1.29 |  |
| Obese | 71 |  | 0.47 | 0.28, 0.80 |  | 47 |  | 0.81 | 0.45, 1.46 |  |
| **Smoking Status** |  | 123 |  |  | **<0.001** |  | 80 |  |  | 0.488 |
| Never smoke | 88 |  | — | — |  | 74 |  | — | — |  |
| <10 pack years | 36 |  | 0.91 | 0.41, 2.03 |  | 32 |  | 0.78 | 0.36, 1.65 |  |
| >= 10 pack years | 209 |  | 2.10 | 1.33, 3.31 |  | 97 |  | 1.17 | 0.72, 1.88 |  |
| **Feeding Tube** |  | 123 |  |  | **<0.001** |  | 80 |  |  | **0.003** |
| No G-tube | 142 |  | — | — |  | 115 |  | — | — |  |
| Temporary G-tube | 116 |  | 1.35 | 0.87, 2.07 |  | 58 |  | 1.06 | 0.62, 1.79 |  |
| Permanent G-tube | 75 |  | 2.71 | 1.75, 4.20 |  | 30 |  | 2.7 | 1.57, 4.66 |  |
| **HPV** |  | 123 |  |  | **0.003** |  | 80 |  |  | **0.087** |
| HPV+ | 52 |  | -- | -- |  | 24 |  | -- | -- |  |
| HPV- | 67 |  | 2.44 | 1.09, 5.49 |  | 32 |  | 1.19 | 0.44,3.22 |  |
| Unknown | 214 |  | 2.93 | 1,42, 6.05 |  | 147 |  | 1.96 | 0.90, 4.29 |  |
| **Tumor site** |  | 123 |  |  | 0.205 |  | 80 |  |  | 0.401 |
| Oral cavity | 163 |  | — | — |  | 143 |  | — | — |  |
| Oropharynx | 89 |  | 0.68 | 0.43, 1.08 |  | 29 |  | 0.83 | 0.44, 1.58 |  |
| Larynx | 54 |  | 1.07 | 0.66, 1.73 |  | 25 |  | 0.54 | 0.25, 1.19 |  |
| Other | 27 |  | 1.29 | 0.69, 2.39 |  | 6 |  | 1.14 | 0.36, 3.66 |  |
| **Charlson Comorbidity Score** |  | 123 |  |  | **0.005** |  | 80 |  |  | **0.03** |
| < 5 | 259 |  | — | — |  | 150 |  | — | — |  |
| >= 5 | 74 |  | 1.78 | 1.21, 2.63 |  | 53 |  | 1.69 | 1.07, 2.68 |  |
| **Pathologic T category (pT)** |  | 121 |  |  | **<0.001** |  | 77 |  |  | **0.002** |
| T0-2 | 208 |  | — | — |  | 146 |  | — | — |  |
| T3-4 | 118 |  | 1.92 | 1.34, 2.75 |  | 52 |  | 2.13 | 1.34, 3.39 |  |
| **Pathologic N category (pN)** |  | 121 |  |  | **0.011** |  | 77 |  |  | 0.336 |
| N0-1 | 203 |  | — | — |  | 145 |  | — | — |  |
| N2-3 | 111 |  | 1.77 | 1.22, 2.56 |  | 40 |  | 1.52 | 0.89, 2.59 |  |
| x | 13 |  | 1.49 | 0.68, 3.26 |  | 13 |  | 1.14 | 0.45, 2.85 |  |
| **Group stage** |  | 121 |  |  | **<0.001** |  | 77 |  |  | **0.002** |
| Stage 1 | 79 |  | — | — |  | 66 |  | — | — |  |
| Stage 2 | 67 |  | 0.84 | 0.43, 1.64 |  | 42 |  | 1.32 | 0.65, 2.69 |  |
| Stage 3 | 49 |  | 1.39 | 0.74, 2.62 |  | 34 |  | 2.87 | 1.50, 5.48 |  |
| Stage 4 | 113 |  | 2.39 | 1.48, 3.87 |  | 56 |  | 2.60 | 1.43, 4.73 |  |
| **Margins status** |  | 110 |  |  | 0.182 |  | 79 |  |  | 0.914 |
| Margins- | 246 |  | — | — |  | 178 |  | — | — |  |
| Margins+ | 59 |  | 1.36 | 0.88, 2.11 |  | 19 |  | 1.04 | 0.50, 2.17 |  |
| **ALI** |  | 109 |  |  | **0.002** |  | 77 |  |  | **0.002** |
| Absent | 177 |  | — | — |  | 147 |  | — | — |  |
| Present | 106 |  | 1.85 | 1.25, 2.73 |  | 33 |  | 2.64 | 1.57, 4.45 |  |
| Indeterminate/Suspicious | 13 |  | 2.61 | 1.18, 5.77 |  | 13 |  | 0.72 | 0.22, 2.29 |  |
| **PNI** |  | 110 |  |  | **0.015** |  | 77 |  |  | **<0.001** |
| Absent | 208 |  | — | — |  | 136 |  | — | — |  |
| Present | 88 |  | 1.64 | 1.11, 2.42 |  | 54 |  | 2.66 | 1.68, 4.21 |  |
| **ENE** |  | 108 |  |  | **0.041** |  | 75 |  |  | 0.833 |
| Absent | 236 |  | — | — |  | 166 |  | — | — |  |
| Present | 57 |  | 1.62 | 1.04, 2.52 |  | 23 |  | 1.08 | 0.54, 2.17 |  |
| **Recurrence** |  | 123 |  |  | **<0.001** |  | 80 |  |  | **<0.001** |
| No recurrence | 253 |  | — | — |  | 149 |  | — | — |  |
| Recurrence | 80 |  | 2.37 | 1.65, 3.39 |  | 54 |  | 3.91 | 2.52, 6.09 |  |
| **RT** |  | 123 |  |  | 0.748 |  | 80 |  |  | 0.706 |
| No RT | 179 |  | — | — |  | 146 |  | — | — |  |
| RT | 154 |  | 0.94 | 0.66, 1.35 |  | 57 |  | 1.10 | 0.68, 1.77 |  |
| **Chemotherapy** |  | 123 |  |  | 0.827 |  | 80 |  |  | 0.819 |
| No Chemo | 261 |  | — | — |  | 176 |  | — | — |  |
| Chemo | 72 |  | 1.05 | 0.68, 1.61 |  | 27 |  | 1.08 | 0.57, 2.04 |  |
| **Treatment Group** |  | 123 |  |  | 0.748 |  | 80 |  |  | 0.554 |
| Surgery | 179 |  | — | — |  | 145 |  | — | — |  |
| Surgery + Adjuvant | 154 |  | 0.94 | 0.66, 1.35 |  | 58 |  | 1.16 | 0.72, 1.85 |  |
|  |  |  |  |  |  |  |  |  |  |  |
| ^1^HR = Hazard Ratio, CI = Confidence Interval | | | | | |  |  |  |  |  |
|  | | | | | | | | | | |

eTable 4.

|  | Sarcopenia | No Sarcopenia | p-value |
| --- | --- | --- | --- |
| **Age (continuous)** |  |  |  |
| **Training set (n = 253)** |  |  |  |
| Men (n = 188) | 62 (55, 69) | 60 (52, 66) | 0.087 |
| Women (n = 65) | 65 (59, 72) | 60 (53, 65) | 0.028 |
| **Validation set (n = 536)** |  |  |  |
| Men (n = 333) | 65 (58, 71) | 61 (54, 69) | 0.013 |
| Women (n = 203) | 65 (57, 73) | 65 (57, 76) | 0.500 |

Supplementary Figure 1. Residual vs fitted value plots for men (n = 188) and women (n = 65). Loess curve is shown in red.


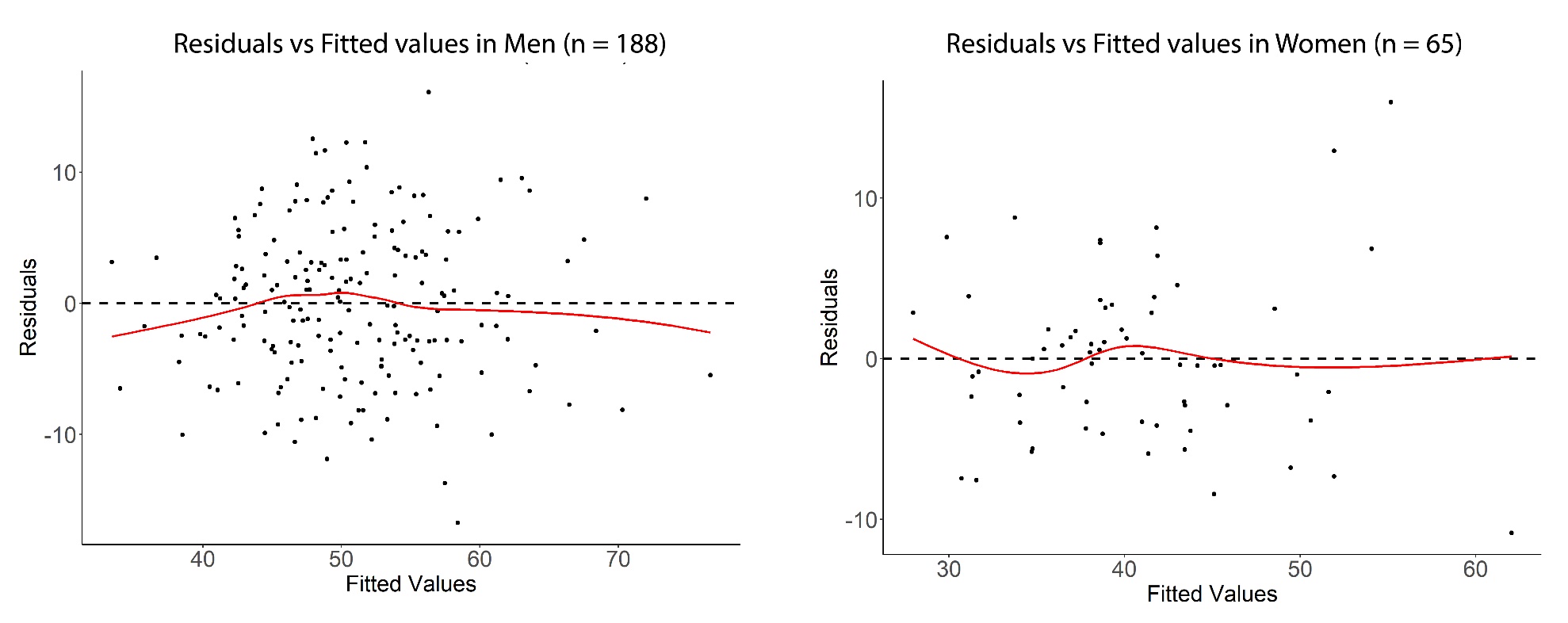


Supplementary Figure 2. ROC curves for L3 and C3 (with and without SCM) in men (n = 188) and women (n = 65).


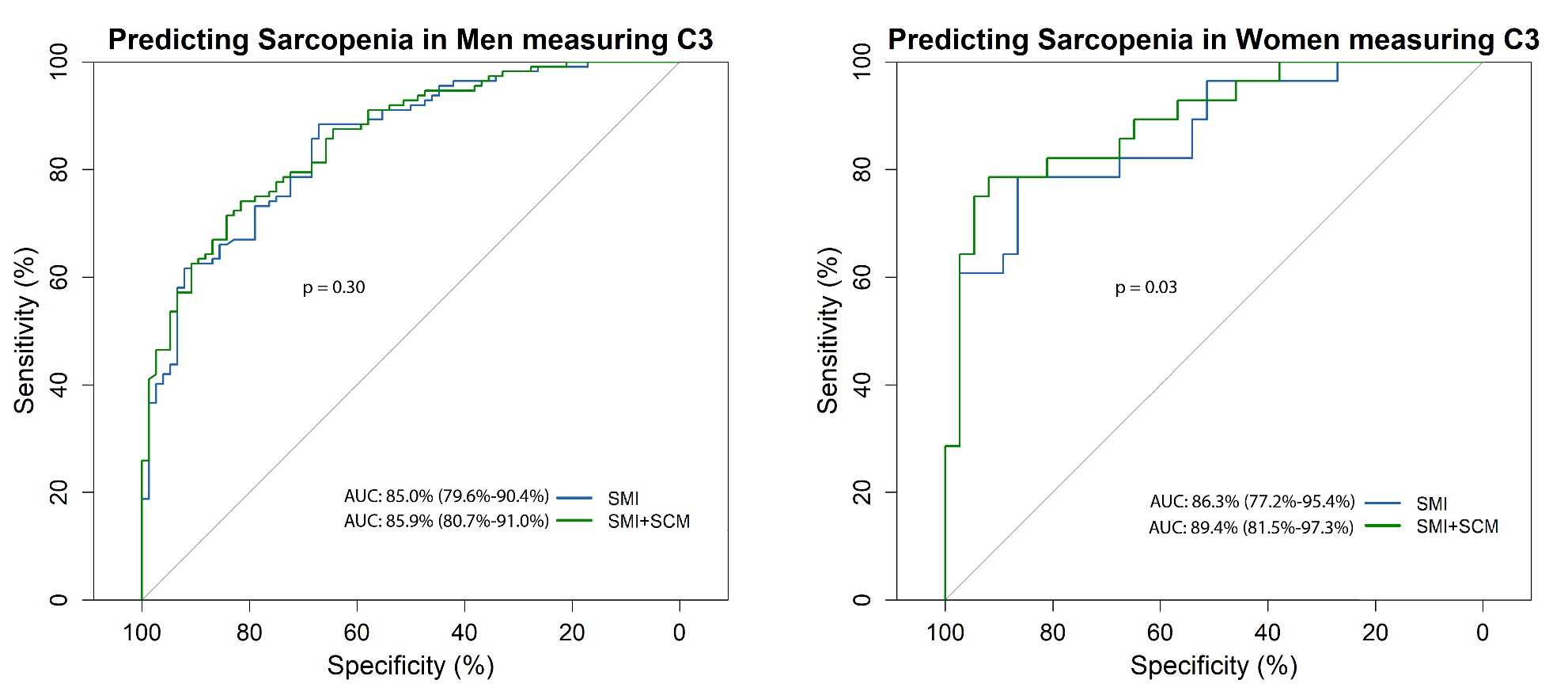


Supplementary Figure 3. Survival analysis in C3-defined sarcopenic male and female HNC patients in the training cohort. Kaplan Meier curves for (A) men and (B) women stratified by C3 sarcopenia status.


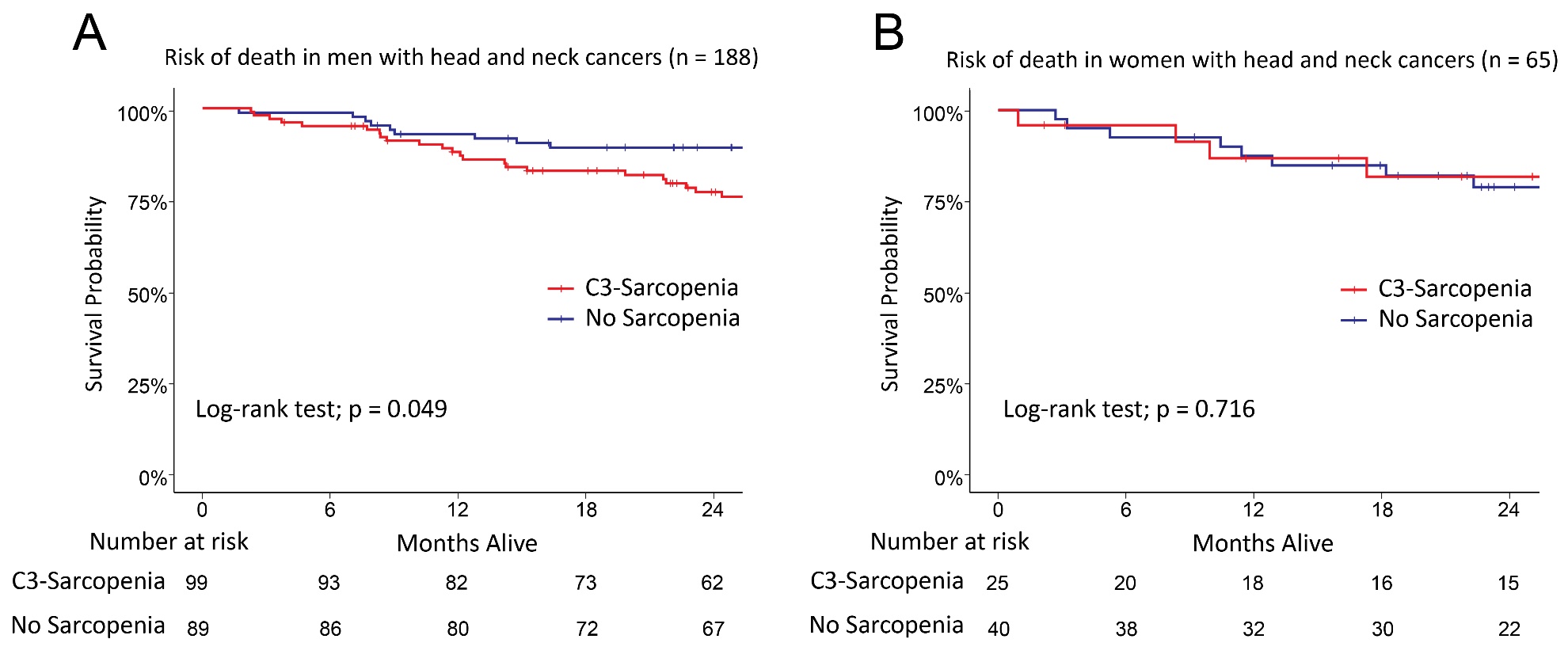

Supplement: Supplementary file 1 [file DataSheet_1.docx]
